# Cinnamic acid shortens the period of the circadian clock in mice

**DOI:** 10.1016/j.bbrep.2016.12.008

**Published:** 2017-01-09

**Authors:** Katsutaka Oishi, Saori Yamamoto, Hideaki Oike, Naoki Ohkura, Masahiko Taniguchi

**Affiliations:** a Biological Clock Research Group, Biomedical Research Institute, National Institute of Advanced Industrial Science and Technology (AIST), Tsukuba, Ibaraki, Japan; b Department of Applied Biological Science, Graduate School of Science and Technology, Tokyo University of Science, Noda, Chiba, Japan; c Department of Computational Biology and Medical Sciences, Graduate School of Frontier Sciences, The University of Tokyo, Kashiwa, Chiba, Japan; d Division of Food Function Research, Food Research Institute (NFRI), National Agriculture and Food Research Organization (NARO), Tsukuba, Ibaraki, Japan; e Molecular Physiology and Pathology, School of Pharma-Sciences, Teikyo University, Itabashi, Tokyo, Japan; f Division of Pharmacognosy, Osaka University of Pharmaceutical Sciences, Takatsuki, Osaka, Japan

**Keywords:** Circadian clock, Cinnamic acid, PER2::LUC protein, Real-time reporter assay, Wheel-running activity

## Abstract

Cinnamic acid (CA) derivatives have recently received focus due to their anticancer, antioxidant, and antidiabetic properties. The present study aimed to determine the effects of cinnamic acid on the circadian clock, which is a cell-autonomous endogenous system that generates circadian rhythms that govern the behavior and physiology of most organisms. Cinnamic acid significantly shortened the circadian period of PER2::LUC expression in neuronal cells that differentiated from neuronal progenitor cells derived from PER2::LUC mouse embryos. Cinnamic acid did not induce the transient mRNA expression of clock genes such as *Per1* and *Per2* in neuronal cells, but significantly shortened the half-life of PER2::LUC protein in neuronal cells incubated with actinomycin D, suggested that CA post-transcriptionally affects the molecular clock by decreasing *Per2* mRNA stability. A continuous infusion of CA into mice via an Alzet osmotic pump under constant darkness significantly shortened the free-running period of wheel-running rhythms. These findings suggest that CA shortens the circadian period of the molecular clock in mammals.

## Highlights

• Cinnamic acid shortened the period of neuronal circadian expression of PER2::LUC.
• Cinnamic acid decreased *Per2* mRNA stability in neuronal cells.
• Cinnamic acid shortened the free-running period of wheel-running rhythms in mice.

## 1 Introduction

The central clock located in the suprachiasmatic nucleus (SCN) of the anterior hypothalamus governs various behavioral and physiological circadian rhythms in mammals such as sleep/wake cycles, body temperature and blood pressure [1]. The molecular mechanism of the circadian clock is cell autonomous and consists of a network of autoregulatory transcription-based feedback loops that drive the rhythmic expression of clock genes such as *Per1* and *Per2*, and these cell-autonomous and self-sustained oscillators are found not only in the SCN but also in peripheral tissues as well as dissociated cells [2], [3]. Functional clock molecules are essential to maintain the normal periodicity of behavioral rhythms [4]. For example, two *Per2* knockout mouse lines have been generated, and the period shortened in both types of mice that subsequently became arrhythmic within 2–3 weeks under constant darkness [5], [6]. A mutation in human *Per2* is associated with familial advanced sleep phase syndrome, an autosomal dominant condition with early morning awakening and early sleep times [7]. Deletion of other clock genes such as *Per1*, *Clock*, *Cry1*, and *Cry2* affects the free-running period of circadian behavior in a gene-specific manner, whereas *Bmal1* knockout results in behavioral arrhythmicity under constant darkness [4]. Acute induction of clock gene mRNA expression plays a critical role in phase shifting the circadian clock *in vivo* and in cells cultured *in vitro*. In addition to transcriptional feedback regulation, other essential roles of post-translational modifications such as phosphorylation and ubiquitination have been identified [8], [9]. Perturbed clock function is implicated in numerous pathologies including sleep disorders associated with circadian rhythms, psychiatric conditions, hypertension, cardiovascular diseases, cancer and metabolic disorders [3].

Cinnamic acid (CA) and its derivatives are abundant in fruits. vegetables and flowers, and have attracted attention due to their anticancer, antimicrobial, antioxidant and antidiabetic properties [10], [11]. Cinnamic acid derivatives such as ferulic acid and cinnamide derivatives also have neuronal effects including anticonvulsant, antidepressant, neuroprotective, analgesic, anti-inflammatory, muscle relaxant and sedative properties [12], [13], [14], [15]. The present study found that CA dose-dependently shortens the circadian period of the molecular clock by monitoring bioluminescence in neuronal cells derived from PER2::LUC mice. We also found that CA significantly shortened the free-running period of behavioral rhythms in mice.

## 2 Materials and methods

### 2.1 Chemicals

(E)-cinnamic acid (>99% pure), luciferin and HEPES were purchased from Wako Pure Chemical Industries, Ltd. (Osaka, Japan). Caffeine (>99% pure), lithium chloride (>99% pure), forskolin, actinomycin D, cycloheximide, penicillin and streptomycin solution were purchased from Sigma-Aldrich (St. Louis, MO, USA). DMEM/F12 and B27 were purchased from Life Technologies (Carlsbad, CA, USA).

### 2.2 Animals and behavioral measurements

Six-week-old male C57BL/6J mice (Japan SLC Inc., Hamamatsu, Japan) were individually housed in cages with SW-15 running wheels (Melquest Ltd., Toyama, Japan) under a 12 h light: 12 h dark cycle at a controlled ambient temperature of 24±1 °C and provided with food and water *ad libitum*. Wheel-running activity was continuously recorded at 5-min intervals using the Chronobiology Kit® (Stanford Software Systems, Stanford, CA, USA) and activity data are displayed as actograms. The free-running period and amplitude of individual mice were estimated using χ^2^ periodgrams.

We obtained neuronal cells expressing PER2::LUC protein from PER2::LUC C57BL/6J knock-in mice (The Jackson Laboratory, Bar Harbor, ME, USA) that were housed as described [16]. Endogenous PER2 protein is fused in-frame with a luciferase reporter in these mice, which allows real-time monitoring of PER2::LUC protein dynamics by recording bioluminescence [17].

This study proceeded in accordance with the guidelines for the Care and Use of Laboratory Animals at the National Institute of Advanced Industrial Science and Technology (AIST), and all procedures were approved by the Animal Care and Use Committee at AIST (Permissions #2013-054 and #2015-020).

### 2.3 Real-time reporter gene assays for neuronal cells

We prepared neuronal cells from day 14 PER2::LUC mouse embryos as described [16]. The cells were stimulated with 10 µM forskolin in differentiation medium (DMEM/F12 containing 2% fetal bovine serum (FBS), B27, 10 units/mL of penicillin and 100 µg/mL of streptomycin) supplemented with 0.1 mM luciferin, 10 mM HEPES and CA at 37 °C. The amount of bioluminescence emitted by PER2::LUC protein was continuously measured and integrated for 1 min at intervals of 15 min with an LM2400 photon detector (Hamamatsu Photonics, Hamamatsu, Japan) that was housed within an incubator maintained at 37 °C. Data were detrended and analyzed using LM2400 software (Hamamatsu Photonics). The time of the second peak was considered as acrophase. The amplitude was averaged over the first four days. Either actinomycin D (1 µM) or cycloheximide (20 µg/mL) and CA were added to neuronal cells in several dishes to determine the rate at which PER2::LUC protein decayed.

### 2.4 Real-time reverse transcription polymerase chain reaction (RT-PCR)

Differentiated neuronal cells were stimulated at time 0 h with either 10 µM CA or with DMSO. The cells were then washed with ice-cold PBS, harvested at the indicated times in RNAiso Plus (Takara Bio Inc., Otsu, Japan) and stored at −80 °C. Total RNA was extracted and then single-stranded cDNA was synthesized using PrimeScript™ RT reagent kits with gDNA Eraser (Takara Bio Inc.). Real-time RT-PCR preceded using SYBR® Premix Ex Taq™ II (Takara Bio Inc.) and a LightCycler™ (Roche Diagnostics, Mannheim, Germany) with the primer sequences as described [18]. The amplification conditions were 95 °C for 10 s followed by 45 cycles of 95 °C for 5 s, 57 °C for 10 s and 72 °C for 10 s. The amount of target mRNA was normalized relative to that of *β-actin*.

### 2.5 Continuous infusion of CA

The mice were continuously housed in the cages with running wheels for at least 20 days under constant darkness. Alzet® model 1004 osmotic mini-pumps (100 μL; 0.11 μL/h; Durect Corporation Cupertino, CA, USA) containing either CA (1 mg/mL) dissolved in 50% DMSO/FBS or vehicle alone were then surgically implanted under dim red light (within 10 min) into the peritoneal cavity of the mice anesthetized with sevoflurane. Skin and muscle incisions were closed with surgical suture. The mice were immediately returned to the cages with running wheels.

### 2.6 Statistical analysis

All results are expressed as means±standard error of the mean (SEM). The data were statistically analyzed using a one-way or two-way ANOVA with Tukey-Kramer or Dunnett’s *post hoc* tests using Excel-Toukei 2010 software (Social Survey Research Information Co. Ltd., Tokyo, Japan). *P*<0.05 indicated a statistically significant difference.

## 3 Results and discussion

**Fig. 1:**
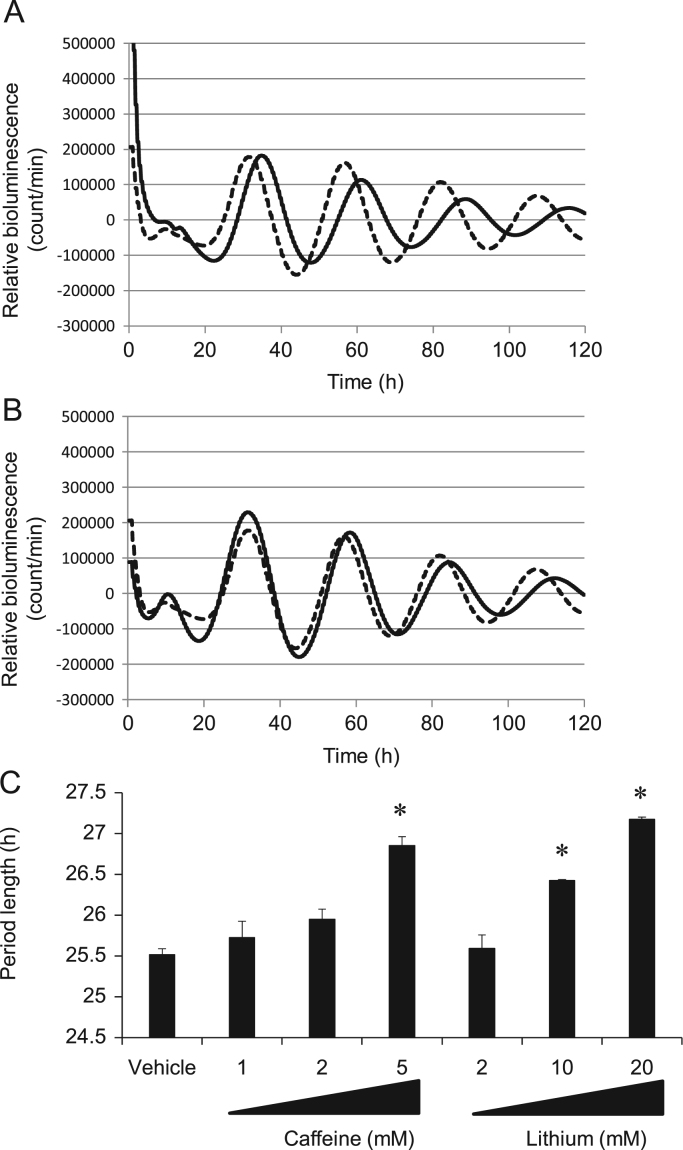
Caffeine and lithium lengthen circadian period of PER2::LUC expression in neuronal cells. (A) Representative detrended data for PER2::LUC neuronal cells incubated with DMSO vehicle (dashed line) or 5 mM caffeine (solid line). (B) Representative detrended data for PER2::LUC neuronal cells incubated with DMSO vehicle (dashed line) or 20 mM lithium (solid line). (C) Effects of caffeine or lithium on period of rhythmic PER2::LUC expression in neuronal cells. Distance between peaks 1 and 3 was multiplied by 0.5 to determine period length. Circadian period of PER2::LUC bioluminescence oscillations was increased by either caffeine or lithium. *P<0.01 vs. vehicle; Dunnett’s test. All values are expressed as means±SEM (n=3 per group).

Many natural and synthetic small molecules are thought to modulate circadian rhythms *in vitro* [19], [20]. Among them, caffeine [21], [22], [23], [24] and lithium [25], [26], [27], [28], [29] affect the period of circadian behavior in rodents and humans. We evaluated whether caffeine and lithium affect rhythmic PER2::LUC activity in neuronal cells derived from PER2::LUC mice in the same manner as they affect mammalian behavior. Both caffeine and lithium dose-dependently lengthened the circadian period of PER2::LUC bioluminescence (Fig. 1), suggesting that neuronal cells expressing PER2::LUC protein can mimic the SCN that governs mammalian behavioral rhythms.

**Fig. 2:**
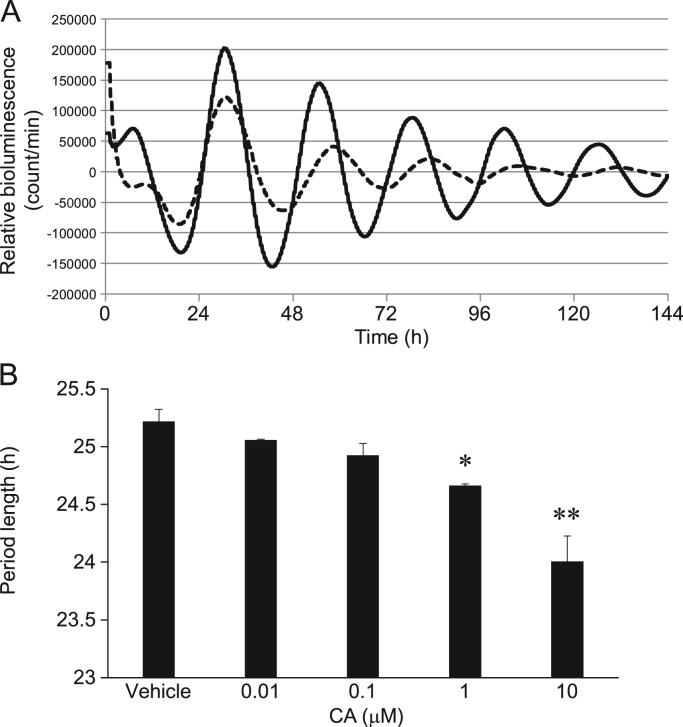
Cinnamic acid (CA) shortens circadian period of PER2::LUC expression in neuronal cells. (A) Representative detrended data for PER2::LUC neuronal cells incubated with DMSO vehicle (dashed line) or 1 µM CA (solid line). (B) Effects of CA on period of rhythmic PER2::LUC expression in neuronal cells. Distance between peaks 1 and 3 was multiplied by 0.5 to determine period length. Circadian period of PER2::LUC bioluminescence oscillation was dose-dependently shortened by CA. *P<0.05, **P<0.01 vs. vehicle; Dunnett’s test. All values are expressed as means±SEM (n=3 per group).

**Fig. 3:**
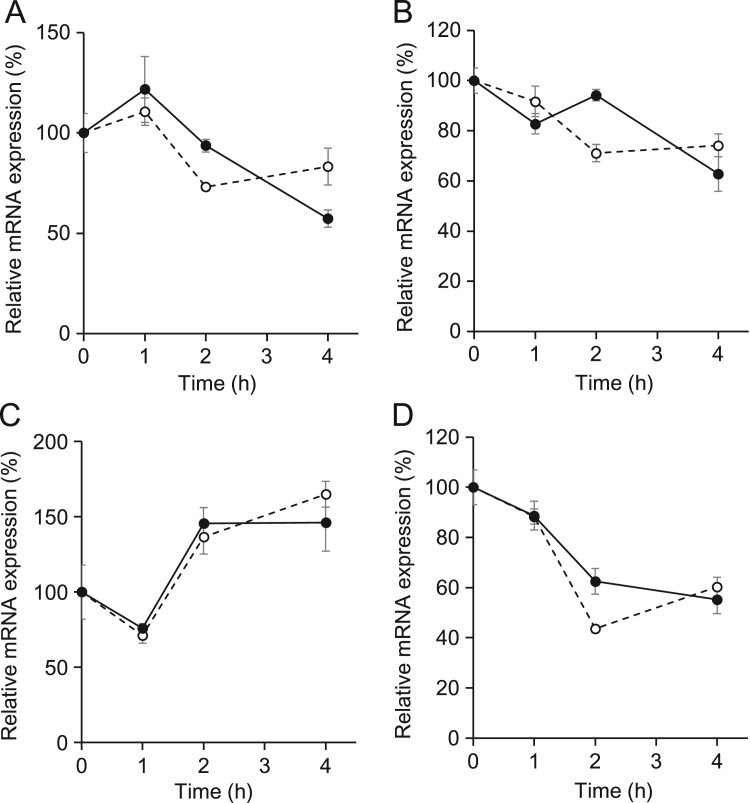
Cinnamic acid (CA) does not induce mRNA expression of clock genes. Expression levels of *Per1* (A), *Per2* (B), *Bmal1* (C) and *Rev-erba* (D) mRNAs assessed by semi-quantitative RT-PCR after stimulation with CA (filled circles) or vehicle (unfilled circles). Values at time 0 are expressed as 100% in each graph. All values are expressed as means±SEM (n=3 – 4 per group).

We measured temporal expression profiles of PER2::LUC in neuronal cells incubated with CA to determine whether CA affects the molecular clock *in vitro* (Fig. 2). Cinnamic acid significantly and dose-dependently shortened the period of PER2::LUC expression (Fig. 2B), and obviously increased the amplitude for >5 days (Fig. 2A). On the other hand, CA did not induce the transient expression of clock genes such as *Per1*, *Per2*, *Bmal1*, *Rev-erbα* in neuronal cells (Fig. 3).

**Fig. 4:**
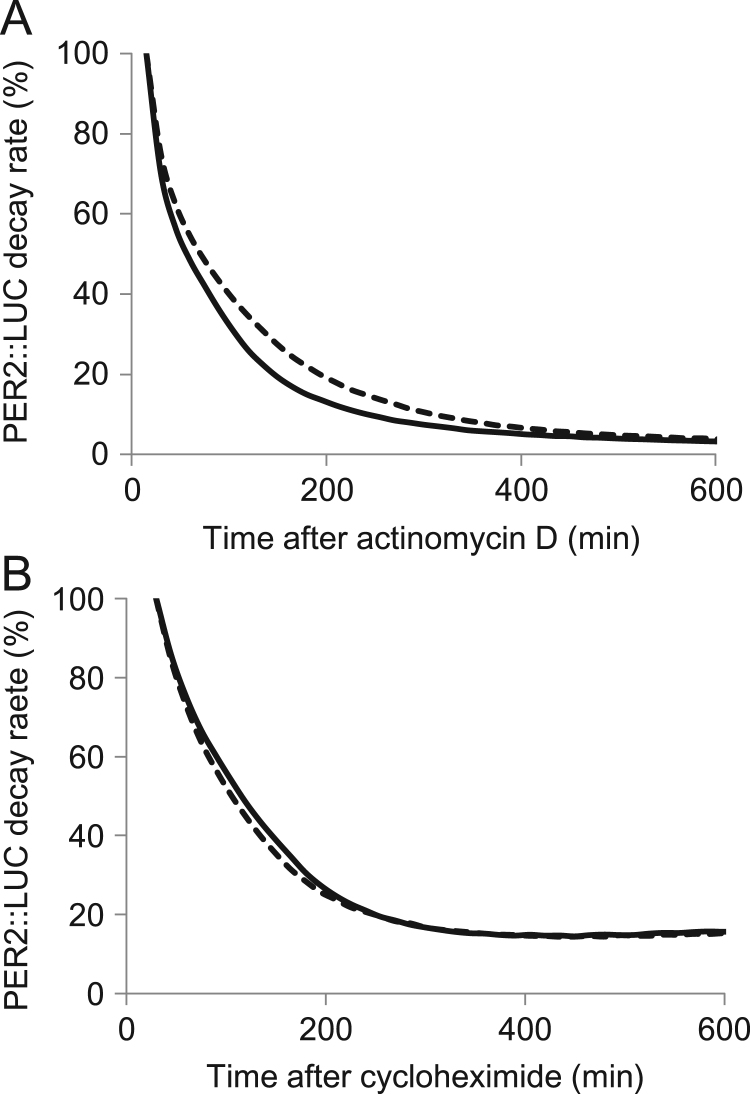
Cinnamic acid (CA) shortens half-life of PER2::LUC protein in neuronal cells incubated with actinomycin D. Representative rate of PER2::LUC protein decay in neuronal cells incubated with DMSO vehicle (dashed line) or 1 µM CA (solid line) and actinomycin D (A) or cycloheximide (B). Half-life of PER2::LUC was determined by normalizing data to time 0 (at actinomycin D or cycloheximide addition) and time 600 (min).

The rate at which PER2::LUC protein decayed was measured in neuronal cells incubated with either actinomycin D (Fig. 4A) or cycloheximide (Fig. 4B) and CA to determine whether CA affects the stability of *Per2* mRNA or PER2 protein. Cinnamic acid significantly shortened the half-life of PER2::LUC protein by 78.8%±2.0% in neuronal cells incubated with actinomycin D (*P*<0.01, n=3; Fig. 3A), but not in those incubated with cycloheximide (106.8%±10.8%, *P*=0.42, n=3; Fig. 3B). These results suggested that CA decreases the mRNA stability of the *Per2* gene. In addition to the transcriptional regulation of clock genes, post-transcriptional mechanisms such as pre-mRNA processing and mature mRNA degradation also play important roles in producing appropriate, rhythmic gene expression in mammals [30], [31]. In fact, *Per2* gene expression appears to largely depend on post-transcriptional regulation [30], [31] compared with other clock genes. Woo et al. [31] demonstrated that 3’-untranslated region (UTR)-dependent mRNA decay is involved in the regulation of *Per2* mRNA circadian oscillation and that polypyrimidine tract-binding protein (PTB) is involved in the stability and degradation kinetics of *Per2* mRNA. They suggested that PTB depletion stabilizes *Per2* mRNA and increases the amplitude of rhythmic *Per2* expression in fibroblasts, although the effect on the circadian period was obscure. On the other hand, LARK binds directly to a cis element in the 3′-UTR of the *Per1* mRNA and stabilizes the mRNA [32]. Knockdown of Lark by siRNA results in a shorter circadian period, and LARK overexpression results in a lengthened period in fibroblasts [32]. Our observations suggest that the enhanced degradation of *Per2* mRNA induced by CA results in shortening the period of the molecular circadian clock, although the underlying mechanism remains unknown.

**Fig. 5:**
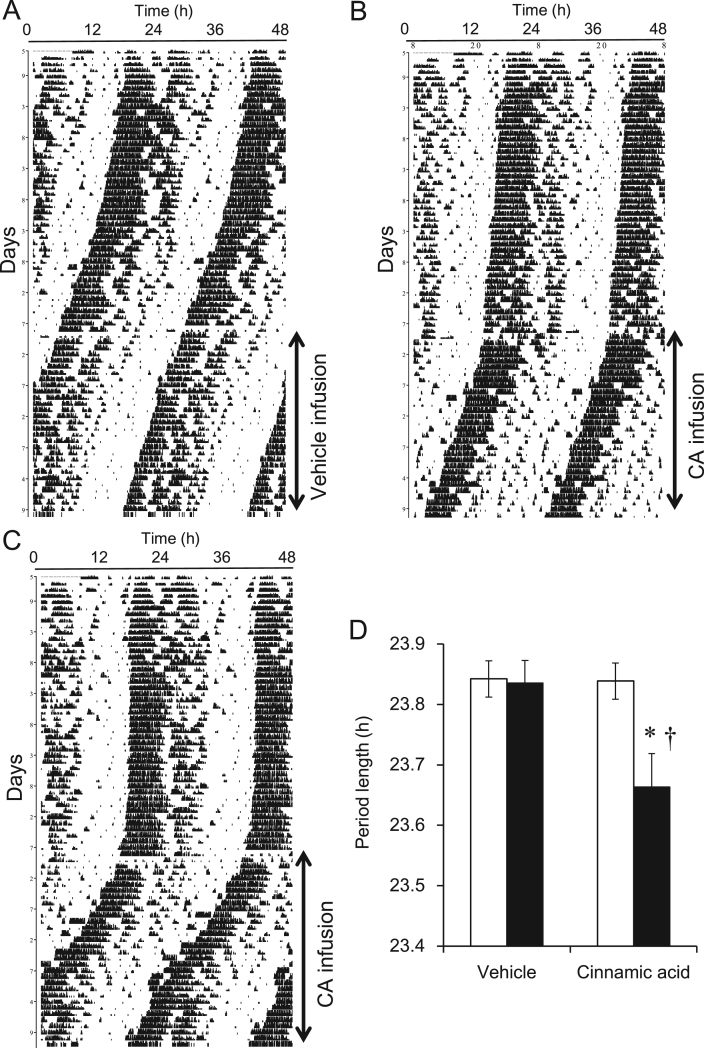
Cinnamic acid (CA) shortens circadian period of wheel-running activity in mice. Mice were continuously housed in cages with running wheels for at least 20 days under constant darkness. DMSO vehicle or 0.1 mg/kg/day CA (0.11 μL/h) was then continuously infused via osmotic mini-pumps. Representative double-plot actograms of wheel running behavior of mice infused with vehicle (A) or CA (B, C). Circadian period of wheel-running activity in mice infused with vehicle or CA before (open bars) and during (filled bars) (D). *P<0.01 vs. before CA infusion (n=13); ^†^*P*<0.05 vs. vehicle infusion (n=15). All values are expressed as means±SEM.

We evaluated the effect of CA on behavioral circadian rhythms by measuring wheel-running activity under constant darkness (Fig. 5). Due to apparently having a relatively short half-life [33], [34], CA (0.1 mg/kg/day) was continuously injected intraperitoneally via mini-pumps to avoid the time-of-day effects of CA on the circadian clock. Before the injection of CA, the periodicity of the mice wheel-running in constant darkness was 23.84±0.03 h. The circadian period slightly, but significantly shortened after CA injection (23.66±0.06 h; Fig. 5D). Vehicle injection did not affect the period. Statistical analysis of the circadian phase and amplitude showed that CA injection did not affect these parameters. These results suggest that CA shortens the circadian period of behavioral rhythms directly or indirectly via the central clock in the SCN. Cinnamic acid and its derivatives have the potential to improve metabolic disorders and might thus exert secondary effects on free-running rhythms through metabolic effects [10], [11]. Cinnamic acid is metabolized *in vivo* to various derivatives that might affect the central clock in the SCN of mice [35], [36]. Cinnamic acid and its derivatives such as ferulic acid comprises one of the largest and most ubiquitous groups of plant metabolites of which many have been structurally identified [10], [11]. Dietary intake of agents enriched with CA derivatives might help to prevent and/or treat exogenous circadian rhythm sleep disorders caused by environmental conditions such as jet-lag or shift-work. Further comprehensive investigations are required to elucidate the underlying mechanisms through which CA modulates the circadian clock.
